# Mycobacterial Phosphatidylinositol Mannoside 6 (PIM_6_) Up-Regulates TCR-Triggered HIV-1 Replication in CD4^+^ T Cells

**DOI:** 10.1371/journal.pone.0080938

**Published:** 2013-11-25

**Authors:** Myriam E. Rodriguez, Candace M. Loyd, Xuedong Ding, Ahmad F. Karim, David J. McDonald, David H. Canaday, Roxana E. Rojas

**Affiliations:** 1 Division of Infectious Diseases, Department of Medicine, Case Western Reserve University, Cleveland, Ohio, United States of America; 2 Department of Molecular Biology and Microbiology, Case Western Reserve University and University Hospitals Case Medical Center, Cleveland, Ohio, United States of America; 3 Cleveland VA Medical Center, Cleveland, Ohio, United States of America; National Institute of Infectious Diseases, Japan

## Abstract

Tuberculosis (TB) is the leading cause of mortality among those infected with human immunodeficiency virus (HIV-1) worldwide. HIV-1 load and heterogeneity are increased both locally and systemically in active TB. *Mycobacterium tuberculosis* (MTB) infection supports HIV-1 replication through dysregulation of host cytokines, chemokines, and their receptors. However the possibility that mycobacterial molecules released from MTB infected macrophages directly interact with CD4^+^ T cells triggering HIV-1 replication has not been fully explored. We studied the direct effect of different MTB molecules on HIV-1 replication (R5-tropic strain Bal) in anti-CD3- stimulated CD4^+^ T cells from healthy donors in an antigen presenting cell (APC)-free system. PIM_6_, a major glycolipid of the mycobacterial cell wall, induced significant increases in the percent of HIV-1 infected T cells and the viral production in culture supernatants. In spite of structural relatedness, none of the other three major MTB cell wall glycolipids had significant impact on HIV-1 replication in T cells. Increased levels of IFN-γ in culture supernatants from cells treated with PIM_6_ indicate that HIV-1 replication is likely dependent on enhanced T cell activation. In HEK293 cells transfected with TLR2, PIM_6_ was the strongest TLR2 agonist among the cell wall associated glycolipids tested. PIM_6_ increased the percentage of HIV infected cells and viral particles in the supernatant in a T-cell-based reporter cell line (JLTRg-R5) transfected with TLR1 and TLR2 but not in the cells transfected with the empty vector (which lack TLR2 expression) confirming that PIM_6_-induced HIV-1 replication depends at least partially on TLR2 signaling.

## Introduction

Tuberculosis (TB) is the largest single cause of death for people living with HIV-1 in low- and middle-income countries, accounting for one-quarter of the estimated 2 million HIV-1 related deaths worldwide [1], [2], [3], [4]. In addition, TB and HIV-1 disease are the two leading causes of infectious disease–associated mortality among adults worldwide [5], [6]. TB is thought to be a major contributor in the immune activation that increases HIV-1 replication, compartmentalization and heterogeneity. Pulmonary TB enhances HIV-1 replication and heterogeneity in the lung [7]. Finally, TB is associated with increased systemic viral replication and heterogeneity, decreased CD4^+^ cell counts, more rapid progression to acquired immune deficiency syndrome (AIDS), and increased mortality [8], [9]. Thus, it has been clearly established that TB has a major impact in viral replication and disease progression in HIV-1 infected individuals. However, the molecular mechanisms that drive HIV-1 disease progression in people with active TB are not well understood.

T cells, especially CD4^+^ T cells, are key to *Mycobacterium tuberculosis* (MTB) infection control. MTB has evolved many strategies to regulate T cell function in order to not only evade immune responses but also promote tissue destruction and transmission. Many of these regulatory loops can affect HIV-1 infected CD4^+^ T cells. For example, pro-inflammatory cytokines secreted by MTB infected macrophages, such as tumor necrosis factor (TNF–α), significantly contribute to the increased viral load observed in HIV-1 infected persons with active TB [4], [10]. Since MTB is an intracellular pathogen, regulation of T cell function by MTB is traditionally considered the indirect result of altered antigen presenting cell (APC) function. Inhibition of antigen processing and presentation, induction of pro-inflammatory or inhibitory cytokines, and control of co-stimulatory molecule expression are MTB mediated mechanisms that regulate APC function and indirectly impact T cell function [11]. However, direct interactions between MTB molecules and T cells may occur when vesicles containing mycobacterial components (exosomes and microvesicles) are released by MTB infected macrophages [12], [13], [14]. Recently, we have shown that MTB proteins and lipoproteins can directly co-stimulate CD4^+^ T cells via TLR2 or integrins [15], [16] and MTB glycolipids can induce T cell adhesion to fibronectin [17]. The role of direct T cell regulation by MTB molecules in MTB/HIV-1 co-infection has not been explored. We propose that mycobacterial molecules released from MTB infected macrophages, interact directly with HIV-1 infected CD4^+^ T cells and trigger virus replication. We tested MTB subcellular fractions and purified glycolipids, which have been reported in exosomes isolated from MTB infected macrophages, for effects on HIV-1 replication in anti-CD3- activated CD4^+^ T cells in an APC-free system. We identified PIM_6_, a mycobacterial cell wall associated glycolipid, as an inducer of HIV-1 replication, increasing the percent of HIV-1 infected T cells and the virus released in culture supernatants. PIM_6_-induced increase in HIV-1 replication correlated with its potent TLR2 agonistic activity and T cell co-stimulatory effects. These results suggest that direct CD4^+^ T cell co-stimulation by MTB molecules may be a major contributor to the increased viral load and accelerated immune dysfunction observed in HIV-1infected individuals with active TB.

## Materials and Methods

### T cell isolation and culture

Human peripheral blood mononuclear cells (PBMCs) were isolated by density gradient centrifugation over Ficoll-Paque™ (GE Healthcare, Uppsala, Sweden) from tuberculin skin test (TST) negative healthy donors (18–45 years old) recruited among laboratory staff. All protocols were approved by Case Western Reserve University institutional review board. Informed written consent was obtained from all participants. Highly pure (>98%) CD4^+^ T cells were obtained from PBMCs by negative selection with mAb coated magnetic beads (Miltenyi Biotec Inc, Auburn, CA). All experiments were performed with purified CD4^+^ T cells cultured in RPMI medium (Fisher Scientific, Pittsburgh, Pa) supplemented with 10% fetal bovine serum (Equitech-Bio Kerrville, TX), 2 mM L-glutamine, 100 U of penicillin, 100 µg of streptomycin and 2-Mercaptoethanol (all from Fisher scientific), 10 mM HEPES, 1X non-essential amino acids mixture and 1 mM sodium pyruvate (all from Lonza, Allendale, NJ).

### MTB subcellular fractions, purified glycolipids and other bacterial products

MTB (H37Rv) fractions and glycolipids from the TBVTRM Collection (NIAID, HHSN266200400091c contract) were provided by the BEI Resources (Manassas, VA). Subcellular fractions were: MTB whole cell lysate (Lys, NR-14822), culture filtrate proteins (CFP, NR-14825) and cell wall fraction (CW, NR-14828). Cell wall associated glycolipids were: phospatidylinositol mannosides (PIM_1_,_2_, NR-14846; PIM_6_, NR-14847), lipomannan (LM, NR-14850) and lipoarabinomannan (LAM, NR-14848). Full-length LprG (rLprG, Rv1411c) was amplified from MTB H37Rv genomic DNA by PCR, cloned in E. coli and expressed in *M. smegmatis* as previously described [18]. The synthetic TLR2 ligand Pam_3_Cys-Ser-(Lys)_4_ (P3CSK4; 2 µg/ml) and the TLR4 ligand purified lipopolysacharide from S. Minnesota (LPS) were from InvivoGen (San Diego, CA).

### Preparation of virus stocks

CCR5-tropic (R5) virus Bal HIV-1 was propagated in U87.CD4.CCR5 cells. Fifty percent tissue culture infectious dose (TCID50) assays were performed to determine virus titer in U87.CD4.CCR5 cells as described [19].

Replication-competent HIV-1 NLAD8 virus stocks were generated by transfection of HEK293T cells with proviral construct pNLAD8 as previously described [20].

### T cell activation and HIV-1 infection

CD4^+^ T cells (0.3×10^6^) were cultured in 48-well plates (Corning® Costar®, Corning, NY) and left untreated (resting) or stimulated with plate bound 10 µg/ml anti-CD3 (clone UCHT1, Ebioscience, San Diego, CA) plus 1 µg/ml soluble anti-CD28 (clone CD28.2 Ebioscience) and anti-CD49d (clone 9F10, Biolegend, San Diego, CA), and 20 U/ml IL-2 (Novartis International AG formerly Chiron Corporation, Emeryville, CA) in medium alone or medium containing MTB molecules. After 2 days at 37°C, cells were transferred to 15 ml conical tubes and infected with Bal HIV-1. Since cells from different donors varied in their susceptibility to viral infection, cells from each donor were infected at two different MOIs (0.1 and 0.01) and data from the MOI condition that rendered the highest infection values were selected for statistical analyses and graphs. Infection was done by centrifugal inoculation at 1200×g for 2 hours at 24°C (spinoculation). After additional hour incubation at 37°C, cells were washed twice with 1X PBS (Fischer) and re-suspended in media. For evaluation of p24 expression by flow cytometry, cells were transferred to polystyrene round bottom tubes and incubated at 37°C for 4 days. For determination of virus in culture supernatants by RT assay cells were transferred to u-bottom 96-well plates and incubated at 37°C for 7 days. Cells treated with 100 µM nevirapine (NVP; catalog no. 4666, AIDS Research and Reference Reagent Program, National Institutes of Health) served as negative control.

### Detection of HIV-1 in CD4^+^ T cells (p24 assay)

The intracellular expression of the *Gag* gene product p24 was determined by flow cytometry. Intracellular p24 expression indicates viral protein synthesis and HIV-1 productive infection. Four days after infection, cells were stained with Live/dead® violet (Invitrogen, Grand Island, NY), anti- human CD4 PerCP (Biolegend, San Diego, CA), anti-human CD3 APC/Cy7 (Biolegend) and anti-human KC57-RD1, which detects 24-kDa HIV-1 capsid antigen (Beckman Coulter Inc., Brea, CA).

Cells were acquired in a LSRII flow cytometer (BD Biosciences, San Jose, CA) and data analyzed with FlowJo software (Tree Star, Inc. Ashland, OR).

### RT assay

Viral replication was monitored by reverse transcriptase (RT) activity in culture supernatants collected at days 3, 5 and 7 post-infection as described previously [21]. Incorporation of [-32P]dTTP by HIV-1 RT is a relative measure of RT activity and virus particles in the supernatant. Donor -to donor variations were detected in the day post-infection at which RT activity peaked. Thus, for each donor the MOI (0.01 or 0.1) and the day of maximum RT activity in culture supernatants was determined and RT values at this time-point were selected for statistical analyses and graphs.

### Detection of IFN-γ in culture supernatants

Supernatants were collected before infection and IFN-γ quantified by sandwich ELISA with the human anti-IFN-γ matched antibody pair M-700A and M-701B, (Thermo scientific, Waltham, MA).

### TLR2 agonistic activity of MTB cell wall associated glycolipids

HEK293 cells stably transfected with human TLR2 and CD14 (293-hTLR2-CD14, InvivoGen) and expressing endogenous TLR1, or the control cell line which does not express TLR2 (293 null, InvivoGen) were incubated in 96-well round bottom plates (20,000 cells/well) for 16 h with LPS, P3CSK4, LprG or MTB glycolipids. In 293/hTLR2-CD14 cells IL-8 secretion as a result of NF-κB activation is a measure of TLR2 stimulation. IL-8 in culture supernatants was quantified using ELISA (R&D, Minneapolis, MN).

### Stable TLR2 transfection of Jurkat T cells

JLTRg-R5 T cells were obtained through the AIDS Research and Reference Reagent Program (Cat #11586). JLTRg-R5 is an enhanced green fluorescent protein (EGFP)-based HIV-1 reporter cell line that expresses CD4, CXCR4 and CCR5 [22]. JLTRg-R5 T cells were stably transfected with TLR1 and TLR2.

Briefly JLTRg-R5 cells were transfected by electroporation (260 V, 960 µF; Gene Pulser II; Bio-Rad) with either control pDuo-mcs plasmids (empty vector) or with pDUO plasmids co-expressing the human TLR1 and TLR2 genes (InvivoGen). After 48 h, cells were plated in serial dilutions in RPMI complete medium containing blasticidin (7.5 µg/ml) and antibiotic-resistant clones expanded for 4–5 weeks. The transfection efficiency was determined by flow cytometry using anti-human CD282 (TLR2, clone 2.5) and anti-human CD281 (TLR1) both from e-Biosciences and confirmed by RNA expression by RT-PCR using TLR1 and TLR2 primers from Invivogen.

### HIV infection/quantification in stable TLR2 transfected Jurkat T cells

JLTRg-R5 transfected with TLR1 and TLR2 vector (JLTRg-R5-TLR1-TLR2) or with empty vector (JLTRg-R5-empty) (0.5×10^6^) were cultured in 48-well plates (Corning® Costar®) and left untreated (resting) or stimulated overnight with anti-CD3 in presence or absence of PIM_6_ or LprG as above. Cells were then infected with NLAD8 HIV-1 for 72 h and the level of HIV infection quantified by measuring p24 intracellular by flow cytometry. In addition, post-infection culture supernatants were collected for RT assay.

### Statistical analysis

Results using different donors are expressed as means (± SEM). The null hypothesis of no difference in means between experimental groups was tested using Student's *t* test. *P* values <0.05 were considered significant.

## Results

### MTB lysate increases productive HIV-1 infection and replication in activated primary CD4^+^ T cells

**Figure 1:**
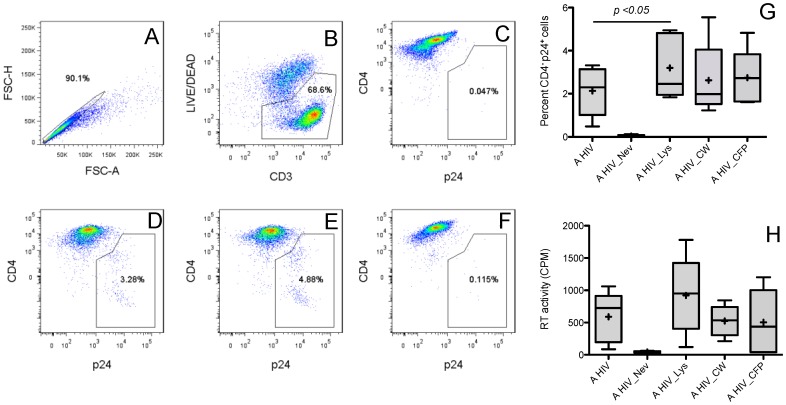
Effect of MTB subcellular fractions on HIV-1 infection in CD4^+^ T cells. **(A–C)** Gating strategy for detection of HIV-1 infected T cells by FACS. CD4^+^ T cells were labeled with live/dead violet, α-CD4 (surface), α-CD3 (surface) and α -p24 (intracellular). Singlet and live cell/CD3^+^ gates were subsequently applied before analyzing CD4 and p24 expression. In resting cells (C), HIV-1 is not detected as demonstrated by absence of CD4^−^ p24^+^ events. **(D–F)** Detection of intracellular HIV-1 by FACS in activated T cells. CD4^+^ T cells were activated with α-CD3, α-CD28, anti-CD49d and IL-2 and with either (D) medium alone or (E) MTB lysate overnight. Cells were then infected with HIV-1 and labeled, acquired and gated as described. **(F)** Cells were treated with nevirapine after infection as a negative control. **(G–H)** Cumulative data for percent of HIV-1 infected T cells (CD4^−^ p24^+^ cells; G) and viral load in culture supernatants (RT assay; H) from five independent experiments with different donors. T cells were infected with HIV-1 (MOI 0.01 to 0.1) after activation with α-CD3, α-CD28/CD49d and IL-2 in medium alone (AHIV) or in medium containing MTB lysate (AHIV_Lys), MTB cell wall (AHIV_CW) or MTB culture filtrate proteins (AHIV_CFP). Cells treated with nevirapine after infection served as negative control (AHIV_Nev). Shown are means (crosses), medians (horizontal lines inside the boxes), first and third quartiles (lower and upper lines of the boxes) and the maximum and minimum values (upper and lower whiskers).

To determine whether MTB molecules could directly affect HIV-1 replication in human CD4^+^ T cells in an APC-independent manner, we used highly purified CD4^+^ T cells (>98% CD3^+^CD4^+^ -data not shown). To avoid potential confounding effects derived from antigen recognition by memory T cells, CD4^+^ T cells were isolated from TST^−^ donors. First, we tested the effect of three MTB crude subcellular fractions, i.e. whole cell lysate, cell wall and culture filtrate proteins on HIV-1 infection in CD4^+^ cells activated with anti-CD3 by measuring intracellular p24 expression by flow cytometry. It is known that the HIV virus type 1 Nef protein triggers CD4 downregulation in primary T cells [23]. To ensure that p24 expression corresponded to productive virus infection and not virus bound to the cell surface we used CD4 down-regulation as a confirmatory sign of virus replication. Thus, increased p24 intracellular staining along with decreased CD4 surface staining indicated productively infected cells. Live single events in the lymphocyte gate were selected for CD4 and p24 expression analysis (Fig.1 A–C). As expected, activated but not resting CD4^+^ T cells supported HIV-1 replication (Fig. 1C–D). Nevirapine, a non-nucleoside reverse transcriptase inhibitor, served as negative control inhibiting productive infection (Fig. 1F). A significantly higher percentage of cells activated in the presence of MTB whole cell lysate was productively infected compared to untreated activated control (Fig. 1E, G). In addition, a modest yet not statistical significant increase in the percentage of productively infected cells compared to control was detected in cells activated in the presence of cell wall fraction or culture filtrate proteins (Fig. 1G).

To confirm active HIV-1 replication in CD4^+^ T cells activated in the presence of MTB subcellular fractions we assessed RT activity in cell-free culture supernatants. RT activity correlates with the number of viral particles and is a sensitive measure of virus production. Predictably, supernatant of resting CD4^+^ T cells showed very low RT activity levels, confirming that HIV replication occurs predominantly in activated CD4^+^ T cells. CD4^+^ T cells treated with MTB whole cell lysate but not with cell wall or culture filtrate proteins showed a trend but not significant higher levels of RT activity compared to untreated activated control cells (Fig. 1H). Thus, both percent of HIV-1 productively infected cells as well as viral production in culture supernatants, indicated that the MTB lysate increases viral replication in activated primary CD4^+^ T cells. These findings using an APC-free system suggest that MTB molecules can stimulate HIV-1 replication by directly interacting with CD4^+^ T cells.

### Identification of PIM_6_ as inducer of HIV-1 replication in activated CD4^+^ T cells

Testing crude MTB subcellular fractions provided preliminary evidence that MTB components can up-regulate HIV-1 replication in CD4^+^ T cells. To identify the factor(s) responsible for up-regulation of HIV-1 replication, we focused on MTB cell wall associated glycolipids because they are known to be associated with exosomes isolated from supernatants of MTB infected macrophages [12], [14]. This ability to traffic outside the infected macrophages makes MTB cell wall glycolipids good candidates for direct contact with CD4^+^ T cells in MTB infection foci where they could trigger HIV-1 replication.

**Figure 2:**
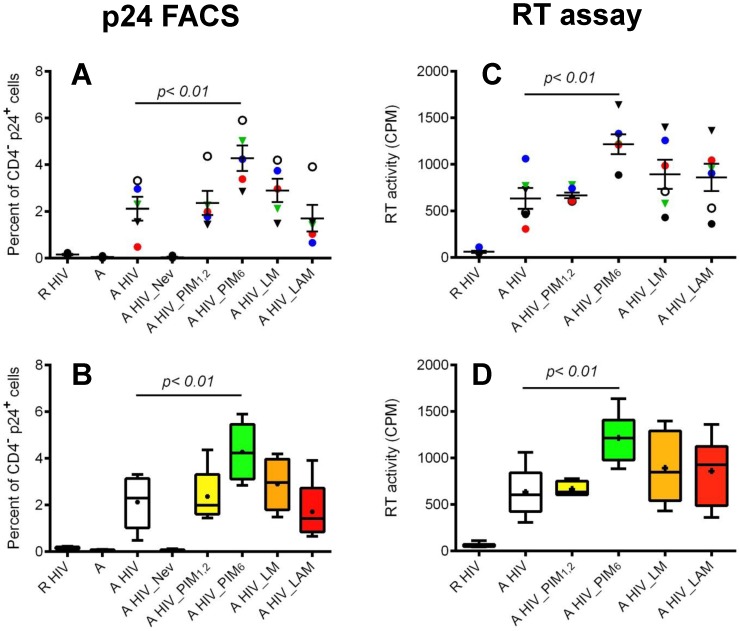
Effect of MTB cell wall associated glycolipids on HIV-1 infection in CD4^+^ T cells. CD4^+^ T cells were infected with HIV-1 at rest (R HIV) or after 48 h activation with α-CD3, α-CD28/CD49d and IL-2 in medium alone (A HIV) or medium containing mycobacterial glycolipids (A HIV_PIM_1,2_, A HIV_PIM_6_, A HIV_LM, A HIV_LAM). Uninfected activated cells (A) served as control. **(A, B)** Intracellular p24 was measured by flow cytometry 4 days after infection. **(A)** Each symbol represents data from one donor and horizontal bars represent means ± SEM. **(B)** Cumulative data from five independent experiments performed with different donors. Shown are means (crosses), medians (horizontal lines inside the box), first and third quartiles (lower and upper lines of the boxes) and the maximum and minimum values (upper and lower whiskers). **(C, D)** Viral load in culture supernatants was determined using the RT assay at day 5 or 7 post-infection. (C) Each symbol represents data from one donor and horizontal bars represent means ± SEM. **(D)** Cumulative data from six independent experiments performed with different donors. Shown are means (crosses), medians (horizontal lines inside the box), first and third quartiles (lower and upper lines of the boxes) and the maximum and minimum values (upper and lower whiskers).

CD4^+^ T cells were activated in the presence of four of the major mycobacterial cell wall associated glycolipids and known to be released in exosomes from MTB infected macrophages, PIM_1,2_, PIM_6_, LM and LAM, and infected with HIV-1. As shown in Fig. 2 A–D, neither the small PIM_1,2_, nor the larger LM or LAM increased significantly the percentage of infected CD4^+^ T cells or the viral load in culture supernatants. By contrast, PIM_6_ significantly increased productive HIV-1 infection in CD4^+^ T cells. PIM_6_ mediated up-regulation of HIV-1 infection was observed in each individual donor with some kinetic variations among donors (Fig. S1). Importantly, PIM_6_ effect on HIV replication were dependent on co-engagement of the TCR, since PIM_6_ had no significant effect in HIV replication in resting CD4^+^ T effects (data not shown). Since the only difference between PIM_1,2_ and PIM_6_ structure is the presence of four additional mannose residues in the latter, our results suggest that up-regulation of HIV-1 replication by PIM_6_ is dependent on a specific interaction between this glycolipid and a receptor on the T cell surface.

### PIM_6_ effect on HIV-1 replication is associated with its T cell co-stimulatory activity

**Figure 3:**
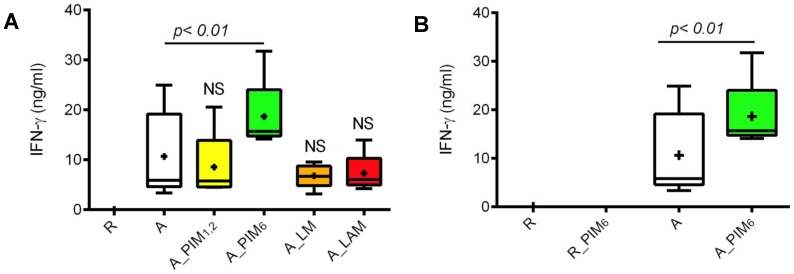
IFN-γ in culture supernatants of activated CD4^+^ T cells in presence of MTB glycolipids. **(A)** CD4^+^ T cells were left unstimulated (resting, R) or were activated with α-CD3, α-CD28/CD49d and IL-2 for 48 h in medium alone (A) or medium containing mycobacterial glycolipids (A_PIM_1,2_, A_PIM_6_, A_LM, A_LAM). **(B)** CD4^+^ T cells were left unstimulated in medium alone (R) or medium containing PIM_6_ (R_PIM_6_) or were activated with α-CD3, α-CD28/CD49d and IL-2 for 48 h in medium alone (A) or medium containing PIM_6_ (A_PIM_6_). IFN-γ in culture supernatants was measured by ELISA. Shown are means (crosses), medians (horizontal lines inside the box), first and third quartiles (lower and upper lines of the boxes) and the maximum and minimum values (upper and lower whiskers) from five independent experiments with different donors.

To examine whether PIM_6_ induced up-regulation of HIV-1 replication is related to T cell activation, we measured IFN-γ production just before HIV-1 infection in supernatants of cells activated in the presence of MTB glycolipids. As shown in Fig. 3, CD4^+^ T cells activated in the presence of PIM_6_ secreted higher levels of IFN-γ compared to control cells activated in medium alone. On the other hand, IFN-γ levels in supernatants of CD4^+^ T cells activated in the presence of PIM_1_,_2_, LM or LAM were not higher than those of control cells. These results demonstrate that the effect of mycobacterial PIM_6_ on HIV-1 replication in CD4^+^ T cells is linked with its effects on T cell co-stimulation. Importantly, PIM_6_ effect on IFN-γ production was dependent on TCR triggering (Fig. 3B). This suggests PIM_6_ engages a co-stimulatory receptor and in this way potentiates the TCR signal, which in turn results in increased activation and permissiveness to HIV-1 infection.

### PIM_6_ is a potent TLR-2 agonist

**Figure 4:**
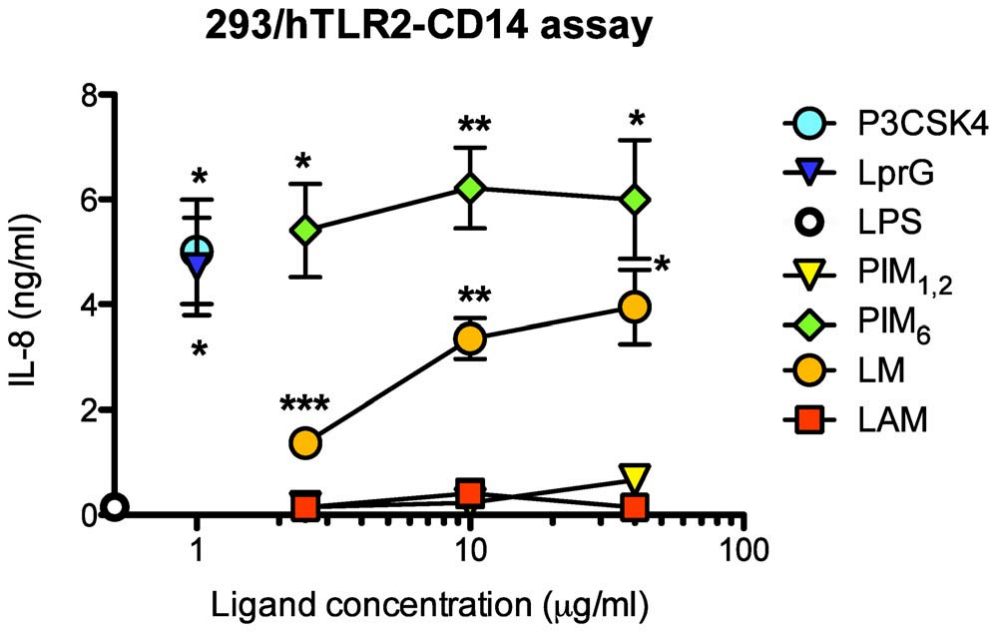
TLR2 agonistic activity of MTB cell wall associated glycolipids. 293-hTLR2-CD14 cells were cultured in 96-well round bottom plates (20,000 cells/well) for 16 h with TLR2 agonist P3CSK4, lipoprotein LprG, TLR4 agonist LPS or MTB glycolipids (PIM_1,2_, PIM_6_, LM, LAM) at indicated concentrations. IL-8 in culture supernatants was quantified by ELISA. Shown are means ± SEM of three independent experiments. * *p*<0.05, ** *p*<0.01, ***p<0.001.

We have recently reported the expression of TLR2 in primary CD4^+^ T cells and a role for mycobacterial TLR2 ligands in CD4^+^ T cell co-stimulation [15]. In addition, others have demonstrated that TLR2 engagement enhances HIV-1 infection in primary CD4^+^ T cells [24], [25], [26]. Thus, to test if PIM_6_ triggers T cell co-stimulation and HIV-1 replication via TLR2, we first determined the relative potency of the four different MTB glycolipids as TLR2 agonists. We used the reporter HEK293 cell line transfected with human TLR2 and expressing endogenous levels of TLR1 (293-hTLR2-CD14) to evaluate the ability of the different glycolipids to engage and trigger TLR1-TLR2. This cell line is also transfected with CD14 to enhance recognition of TLR2 ligands and increase sensitivity of TLR2 responses. We found that while PIM_1_,_2_ and LAM had no TLR2 agonistic activity, LM and PIM_6_ triggered signaling that resulted in the NF-κB dependent transcription of IL-8 (Fig.4). IL-8 secretion by 293-hTLR2-CD14 cells was also observed in response to two model TLR2 agonists, i.e. mycobacterial lipoprotein LprG and synthetic lipopeptide P3CSK4, used as positive controls of TLR2 engagement and signaling. Also, LPS did not induce IL-8 secretion, confirming PIM_6_ and LM effects are not mediated by TLR4. Neither LM nor PIM_6_ induced IL-8 secretion from a control cell line not expressing TLR2 (not shown). Thus, IL-8 secretion triggered by PIM_6_ and LM was dependent on the expression of TLR2. PIM_6_ was a more potent ligand for TLR2 than LM on a molar basis (Fig. 4, compare responses to LM 10 µg/ml = 1.81 µM to PIM_6_ 2.5 µg/ml = 0.96 µM). These results indicate that PIM_6_ is the strongest TLR2 ligand among the main four species of mannosylated glycolipids found in the mycobacterial cell wall. This suggests that PIM_6_ TLR2 agonistic activity may be responsible for the T cell co-stimulatory effect that likely triggers HIV-1 replication in CD4^+^ T cells.

### PIM_6_ up-regulates HIV-1 infection via TLR2

**Figure 5:**
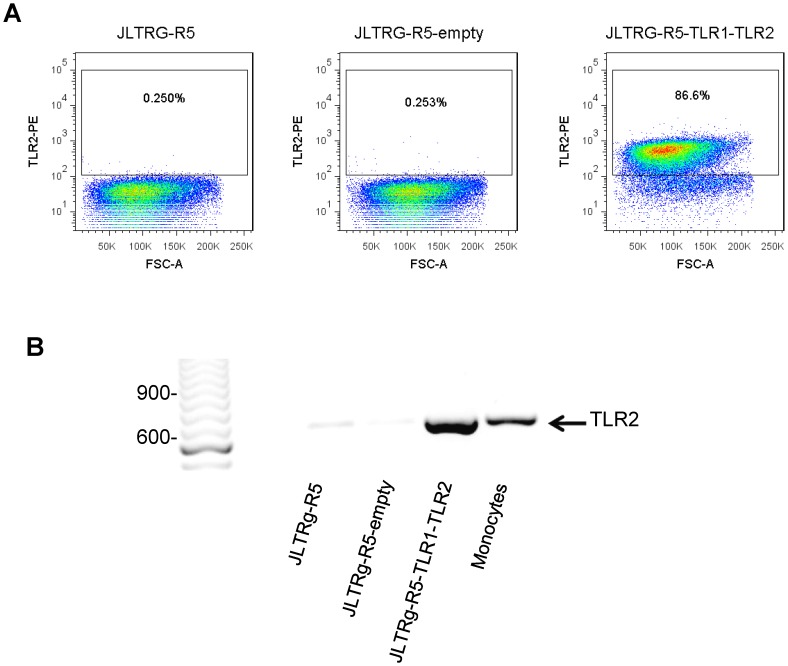
TLR2 expression on TLR1-TLR2 transfected Jurkat JLTRg-R5 T cells. **(A)** TLR2 protein expression on JLTRg-R5 cells before (JLTRg-R5) and after transfection with empty vector (JLTRg-R5-empty) or with vector encoding TLR2/1 (JLTRg-R5-TLR1-TLR2) was determined by flow cytometry. **(B)** Expression of TLR2 mRNA in JLTRg-R5 before (JLTRg-R5) and after transfection with empty vector (JLTRg-R5-empty) or with vector encoding TLR2/1 (JLTRg-R5-TLR1-TLR2) was measured by RT-PCR. Primary monocytes (MN) served as positive control. Shown is one representative experiment of three.

**Figure 6:**
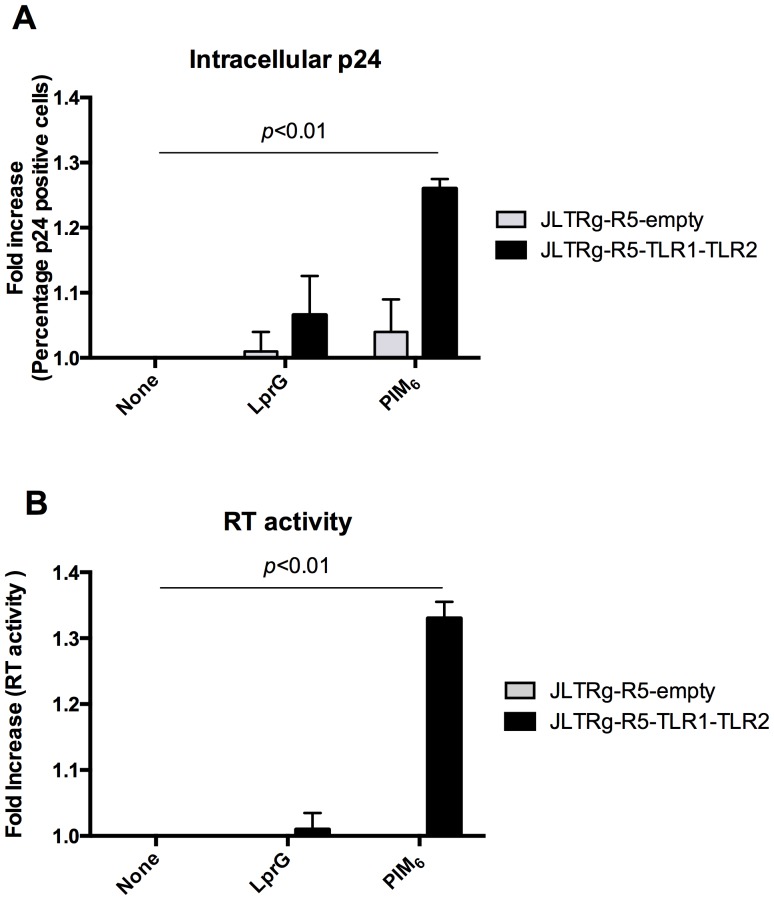
PIM_6_ up-regulates HIV-1 infection via TLR2. JLTRg-R5-TLR1-TLR2 and JLTRg-R5-empty were activated overnight with α-CD3 in medium alone (None) or medium containing LprG (LprG) or PIM_6_ (PIM_6_). Cells were then infected with NLAD8 virus for 72 h and viral replication determined by measuring intracellular p24 by flow cytometry (**A**) or RT activity in culture supernatants (**B**). **(A)** Shown are means ± SD of four independent experiments. **(B)** Shown are means ± SD of two independent experiments in triplicate.

We next assessed if PIM_6_-triggered enhancement of HIV-1 replication in T cells was mediated by TLR2. Since blocking antibodies and siRNA are only partially effective at blocking TLR2-ligand interaction or knocking down TLR2 expression respectively, we decided to express TLR2 in an HIV reporter Jurkat T cell line, i.e. JLTRg-R5 cells, that lacks TLR2 expression both constitutively and after activation (Fig. 5 A, B). Transfection of JLTRg-R5 T cells with the TLR1-TLR2 construct resulted in TLR2 expressing cells as measured by flow cytometry and confirmed by RT-PCR. Activation of TLR2^+^ JLTRg-R5 T cells (JLTRg-R5-TLR1-TLR2) in the presence of PIM_6_ resulted in increased HIV-1 infection as measured by intracellular p24 expression compared to cells activated in medium alone (Fig 6-A). On the other hand, PIM_6_ had no effect on HIV infection in activated JLTRg-R5 T cells transfected with empty vector (JLTRg-R5-empty). Albeit not statistically significant, a trend towards an increase in HIV infection was also observed when JLTRg-R5-TLR1-TLR2 cells were activated in the presence of the mycobacterial TLR2 ligand LprG.

These results were confirmed by RT assay that demonstrated increased virus released in culture supernatants from JLTRg-R5-TLR1-TLR2 T cells but not from JLTRg-R5-empty T cells activated in the presence of PIM_6_ (Fig 6-B). Since the strong TLR2 agonist LprG did not have significant effects on HIV replication, it is conceivable that PIM_6_ effects on HIV infection are mediated by TLR2 in combination with engagement of another unidentified receptor. However, these data suggest that TLR2 engagement is at least partially responsible for PIM_6_ effects on HIV infection in CD4^+^ T cells.

## Discussion

The primary targets of regulation of MTB and other intracellular pathogens are APCs, i.e. macrophages and dendritic cells (reviewed in [11]). However, microvesicles and exosomes containing mycobacterial glycolipids and lipoproteins can traffic outside the infected APC affecting the function of uninfected neighboring cells and expanding the regulatory reach of MTB [12], [14], [27], [28]. Our previous studies demonstrated that CD4^+^ T cells can interact with several MTB molecules directly without APC intervention [15], [16], [17]. These direct effects of mycobacterial molecules on T cells provide novel mechanisms that, in addition to cytokines and chemokines, may explain how MTB promotes HIV replication in co-infected people. In this study we analyzed the direct effect of mycobacterial cell-wall associated glycolipids in HIV-1 infection of primary CD4^+^ T cells.

MTB's cell wall has a high content of mannosylated glycolipids including PIMs, LM and LAM, which are important factors in the immunopathogenesis of TB. PIMs, LM and LAM are biosynthetically and structurally related, and share a conserved phosphatydinositol (PI) anchor with mannosylation extension at the C-6 position of the myo- inositol. The major PIM species are PI-dimannosides (PIM_2_) and PI-hexamannosides (PIM_6_). In addition to the PI anchor, LM and LAM possess a mannan core composed on average of 20–25α (1→6)-linked Man*p* residues occasionally substituted at C-2 by single Man*p*. In the case of LAM, a branched arabinan polymer is attached to the mannan core and cap motifs attach to the non-reducing termini of the arabinosyl side chains [29]. In spite of their structural relatedness, our results indicate that PIM_6_ is unique among mycobacterial mannosylated glycolipids in its ability to increase HIV-1 infection in primary CD4^+^ T cells and this is at least partially related to its ability to engage and signal via TLR2.

TLR2 is mainly expressed on innate immune cells such as macrophages, dendritic cells and NK cells [30], [31]. However, TLR2 has also been reported on T cells where it functions as a co-stimulatory receptor [15], [32], [33], [34], [35]. *In vivo* and *in vitro* studies have provided evidence that TLR2 ligands promote HIV-1 replication in T cells. These reports included both indirect mechanism mediated by APCs [26], [36], [37], [38], [39] and direct effects on T cells [24], [25], [40]. Most of these studies have used synthetic lipopeptides based on the structure of natural bacterial lipoproteins [24], [25], [40]. To our knowledge this is the first report of a mycobacterial glycolipid directly triggering HIV-1 replication in CD4^+^ T cells via TLR2 in an accessory cell independent manner.

PIM, LM and LAM have diverse effects on the immune system. PIMs but not LM or LAM, induces T cell adhesion to fibronectin via VLA-5 [17]. LM is a potent TLR2 agonist and triggers pro-inflammatory and microbicidal innate immune responses [41], [42], [43]. On the other hand, likely due to the presence of the arabinan domain, LAM is less able to engage and activate TLR2 [43]. The ability of PIMs to engage and trigger signaling via TLR2 still remains controversial [29], [44]. Our data demonstrates that PIM_6_ but not PIM_1_,_2_ triggers TLR2 signaling in a reporter HEK cell system. Crystallographic data of the TLR2/TLR1 heterodimer bound to P3CSK4 indicated that the three lipid chains of this tri-acylated lipopeptide mediate the heterodimerization of the receptor with the two ester-bound lipid chains inserted into a pocket in TLR2, and the amide-bound lipid chain inserted into a hydrophobic channel in TLR1 [45]. Both PIM_1_,_2_ and PIM_6_ consist of a mixture of tri- and tetra-acylated species and thus, have presumably the same TLR1-TLR2 binding capacity. This suggests that, while both PIM_1,2_ and PIM_6_ may bind TLR1-TLR2 via their acyl groups, only PIM_6_ triggers signaling via this receptor. Thus, we propose that PIM_6_ terminal mannosyl units may be required for either triggering receptor conformational changes or for binding to an accessory receptor required for signaling [18]. Although CD14 is known to serve as an accessory receptor for TLR2 ligand binding in APCs, this receptor is not expressed on T cells. Accordingly, it is conceivable that an accessory receptor different from CD14 remains to be identified in T cells. The reason why LM being a strong TLR2 agonist in the HEK293 assay, does not co-stimulate or significantly increase HIV-1 infection in primary CD4^+^ T cells may be related to a dominant TCR inhibitory effect of this glycolipid. We have reported that higher order glycolipids such as LAM [46] and LM (Rojas, unpublished observations) have the ability to insert into T cell membranes and inhibit early TCR signaling. Our IFN-γ production data support this statement since IFN-γ levels in supernatants of CD4^+^ T cells activated in the presence of LM or LAM was not higher than those of control cells. A lower TCR signaling will ultimately lead to lower T cell activation and HIV-1 infection due to lack of co-receptors and transcription factors required for viral entry and transcription. Therefore, considering the divergent direct effects of MTB glycolipids on CD4^+^ T cells, we speculate that changes in the mycobacterial glycolipid content of exosomes and microvesicles released from MTB infected macrophages may have a role in the fine regulation of HIV-1 replication in the MTB infectious foci. Exosomes and microvesicles carrying an excess of PIM_6_ could lead to viral replication while exosomes and microvesicles carrying higher order LAM or LM may have no effect on viral replication. It is possible that the exosome and microvesicles composition varies with the mycobacterial strain or with host factors such as cytokines or metabolic products and this may contribute to the progression of HIV-1 infection in co-infected individuals. We are currently conducting follow up studies on exosome biogenesis during MTB infection, characterizing changes in exosome composition in response to different stimuli and studying how these changes impact regulation of CD4^+^ T cell function and HIV replication

Although experiments with Jurkat T cells transfected with TLR2 demonstrate that PIM_6_ effect on HIV-1 infection of T cells depends at least in part on this receptor, co-engagement of a second receptor cannot be ruled out. This is suggested by the lack of significant effect of LprG, a strong TLR2 agonist. We have previously reported that PIM_6_ binds to the beta1 integrin VLA-5 [17]. Like TLR2, VLA-5 functions as a co-stimulatory receptor using the focal adhesion kinase (FAK) pathway to provide TCR complementary signaling [17], [47],[48]. Both FAK and TLR2 signaling result in NF-κB activation and its nuclear translocation [49], [50], [51], [52], [53]. Since NF-κB has been shown to regulate viral transcription via the two NF-κB sites in the HIV-1 LTR [54], NF-κB activation and nuclear translocation provides a mechanism by which PIM_6_ may increase HIV-1 replication in primary CD4^+^ T cells. However, T cell co-stimulation may also lead to increased HIV co-receptor expression and activation of other transcription factors such as NF-ATc that could provide additional pathways for up-regulation of HIV-1 infection in CD4^+^ T cells interacting with PIM_6_ [25], [55]. Thus, the effect of PIM_6_ on HIV-1 replication in CD4^+^ T cells may be mediated by binding of both TLR2 and VLA-5, which in turn may trigger synergistic intracellular signaling pathways. Further studies will be required to better understand the involvement of VLA-5 and to identify the intracellular pathways and step(s) in the viral life cycle promoted as a result of PIM_6_ direct interaction with CD4^+^ T cells.

Considering that monocytes/macrophages may constitute an HIV reservoir [56], [57] and MTB glycolipids traffic inside infected macrophages, it is conceivable that, in addition to inducing HIV-1 replication in T cells, PIM_6_ could also induce HIV replication in macrophages. These effects could take place in cis (HIV/MTB co-infected macrophages) or trans (MTB-infected macrophage to HIV-infected bystander macrophage) within an MTB granuloma. In a separate study, we have demonstrated PIM_6_ induces HIV-1 latency reactivation in monocyte/macrophage cell lines (Alvarez-Carbonell, Rojas et al., unpublished). Thus PIM_6_ may affect the HIV life cycle in different cell types and at different stages of HIV infection (acute, latent) contributing to the clinical decline of MTB/HIV co-infected individuals.

Due to the focused nature of our screening we cannot rule out the possibility of MTB molecules other than cell wall associated glycolipids being partially responsible for the effects observed with whole MTB lysate. Comprehensive screenings of MTB fractions are in process to test this possibility.

In conclusion, we identified for the first time that PIM_6_, a major cell wall associated mycobacterial glycolipid, up-regulates HIV-1 productive infection in primary CD4^+^ T cells in a direct manner that is independent of accessory cells. Direct regulation of T cells by mycobacterial molecules actively secreted by MTB infected macrophages may have a role in triggering viral replication at the site of MTB infection.

## Supporting Information

Figure S1 **Kinetics of HIV-1 infection in CD4^+^ T cells activated in presence or absence of PIM_6_.** CD4^+^ T cells were infected with HIV-1 after 48 h activation with α-CD3, α-CD28/CD49d and IL-2 in medium alone (A HIV) or in medium containing 40 µg/ml of PIM_6_ (A HIV_PIM_6_). Viral load in culture supernatants was determined using the RT assay at day 3, 5 and 7 post-infection. Each panel represents kinetic of HIV infection in CD4+ T cells from a single donor at different time-points. Shown are means ± SD of triplicates.
(TIF)
