# Supplementary figures and images for: Mycobacterial Phosphatidylinositol Mannoside 6 (PIM6) Up-Regulates TCR-Triggered HIV-1 Replication in CD4+ T Cells
Source: PLoS One. 2013 Nov 25;8(11):e80938. doi: 10.1371/journal.pone.0080938 (PMC3839890; doi:10.1371/journal.pone.0080938)

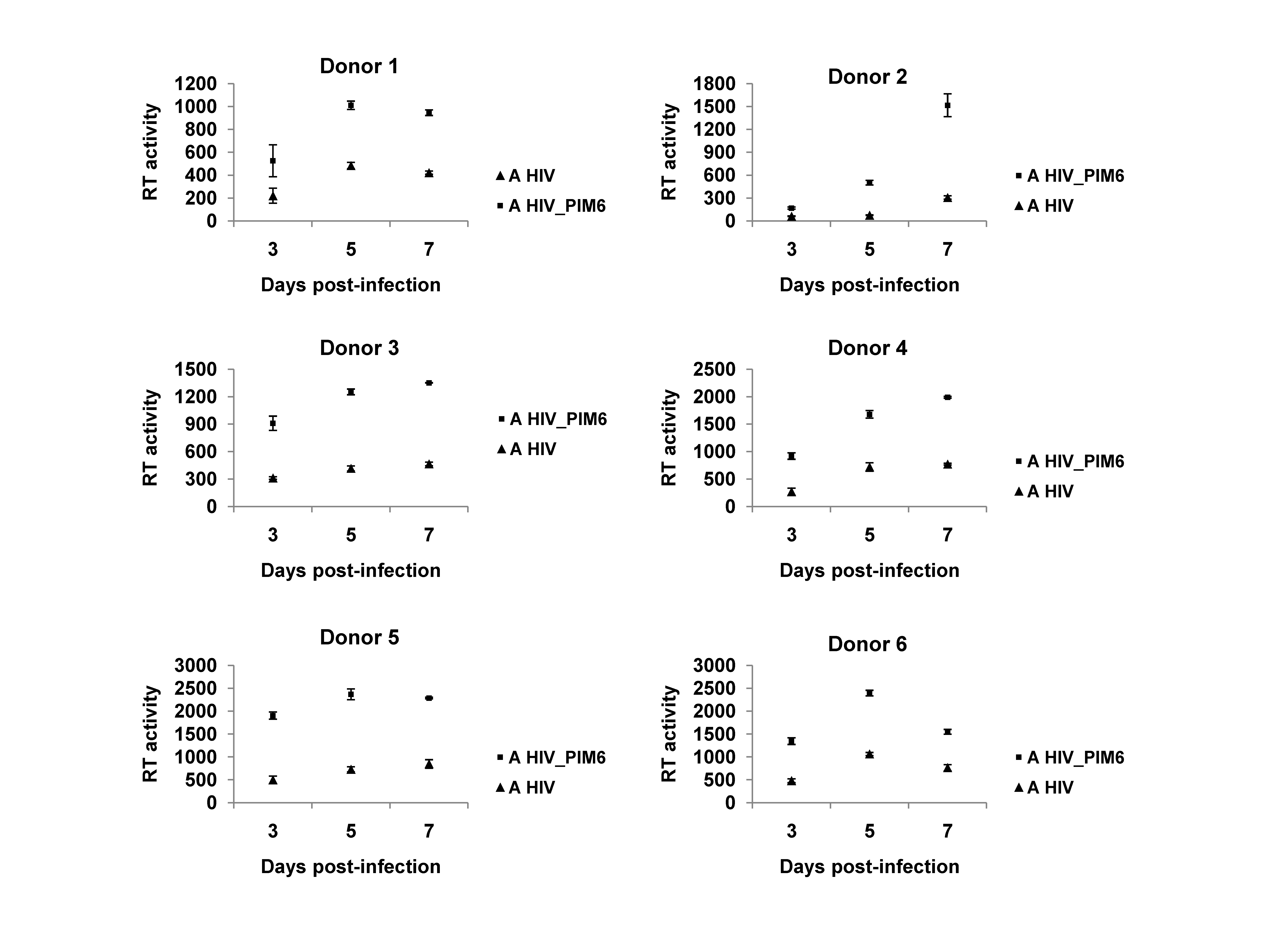

Supplement: Figure S1 — Kinetics of HIV-1 infection in CD4+ T cells activated in presence or absence of PIM6. CD4+ T cells were infected with HIV-1 after 48 h activation with α-CD3, α-CD28/CD49d and IL-2 in medium alone (A HIV) or in medium containing 40 µg/ml of PIM6 (A HIV_PIM6). Viral load in culture supernatants was determined using the RT assay at day 3, 5 and 7 post-infection. Each panel represents kinetic of HIV infection in CD4+ T cells from a single donor at different time-points. Shown are means ± SD of triplicates. (TIF) [file pone.0080938.s001.tif]
